# Mobile COWs (Computer on Wheels): Hamburger or VEAL?

**DOI:** 10.5811/westjem.2016.6.30118

**Published:** 2016-07-20

**Authors:** Maxwell Jen, Tiffany Cho, Scott Rudkin, Andrew Wong, Negin Almassi, Erik Barton

**Affiliations:** * University of California, Irvine Medical Center, Department of Emergency Medicine, Orange, California; † University of California, Irvine Medical School, Department of Emergency Medicine, Irvine, California

## INTRODUCTION

The HITECH (Health Information Technology for Economic and Clinical Health) Act of 2009 galvanized the universal adoption of electronic health record (EHR) systems to improve the quality, delivery, and coordination of patient care.^1^ Initial results demonstrated improvement in population health outcomes and increased transparency.^2^–^3^ Through the HITECH Act’s Meaningful Use (MU) incentives, EHR adoption also promised shorter hospital stays, reduced costs and improved access to healthcare data.^4^ These promises, however, never materialized; studies have demonstrated that EHR adoption causes decreased rates of patients seen per hour, highly variable documentation times, and increased order entry times.^5^

The unintended consequences of the HITECH Act are exacerbated in the emergency department (ED). While the few studies examining practical limitations of ED EHR use are limited to single-site studies with variable, non-validated outcomes, they suggest that MU obstructs ED best customs and practices and is potentially dangerous. 6–7 For instance, real-time computerized charting is difficult because it requires a bedside computer and Internet access, but installing the required hardware is limited by cost and regulations governing the use and renovation of hospital facilities.^8^ MU requirements also stipulate a transition to computerized physician order entry (CPOE); however, prior studies have demonstrated that CPOE increases order entry times, exacerbating the well-documented issue of ED crowding and boarding.^1^,^5^,^9^ In emergent situations, CPOE forces physicians to leave the deteriorating patient’s bedside to access a computer before treatment can be rendered.

## REVIEW OF CURRENT TECHNOLOGY

Mobile technology could solve these issues: physicians remain at the bedside, no hardware installation other than a Wi-Fi router would be needed, and healthcare facilities would be in compliance with MU. Unfortunately, a market survey of commercially-available EHR systems demonstrates that none have effective mobile platforms; one study comparing the usability of several mobile EHR products found that all ranked below a system usability scale (SUS) of 68 (a score considered average).^10^–^11^

Some facilities have adopted Computers-On-Wheels (COW) as mobile workstations.^12^ A market survey demonstrates that most commercially-available mobile workstations are expensive, frequently costing over $3,000 per unit. This does not include the cost of the computer or accessories. Additionally, the COW’s hefty weight and footprint precludes effective mobility especially when multiple units are in use. Imagine the all-too-familiar situation in which nurses, technicians, and physicians are each trying to perform their patient care tasks in the treatment room, but now with COWs in tow. Furthermore, once spent, the COW’s battery must be recharged at a power outlet or charging station rendering the COW immobile for several hours. Lastly, it may be challenging for many EDs to find the approved physical space necessary to store and charge a row of multiple COWs.

The purpose of this paper is to describe the development and initial implementation of a potentially superior mobile computing solution in a single prototypical ED.

## METHODS

**Figure 1:**
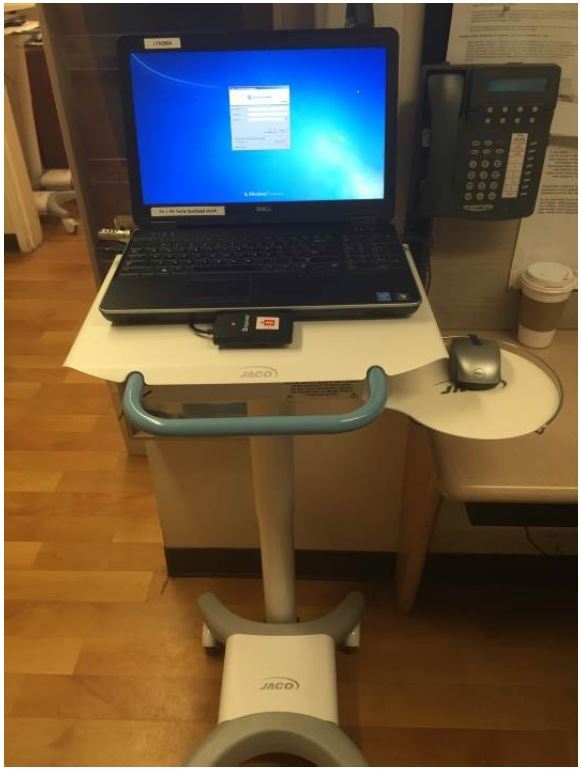
The Very-Efficient Agile Laptop (VEAL).

Development of our mobile computing solution dubbed the *Very-Efficient Agile Laptop* (VEAL) began in January 2015 and lasted three months (Figure 1). It is, in summary, a laptop computer mounted to a mobile workstation. While a variety of parameters were considered in the VEAL’s design, three features were considered functionally critical:

### Compact footprint

Since the COW’s size limited efficient mobility and storage, careful consideration was given for the VEAL’s footprint. The design team chose to mount a *Dell Latitude E6450* laptop atop an *UltraLite 200 Series, 200 Model* mobile podium produced by *JACO Inc.* The laptop measures 14.92 × 1.31 × 9.86 inches and weighs 5.64 pounds, while the podium maintains a footprint of 20 × 16 inches with an adjustable height of 30 to 46 inches and weighs 44 pounds.

### Wi-Fi Video Conferencing Capability

While Wi-Fi access is required for basic access and use of the EMR, using the laptop’s built-in video camera also affords the use of translation services, including American Sign Language (ASL), as well as telehealth, e.g. tele-neurology and tele-psychiatry services.

### Exchangeable Batteries

**Figure 2:**
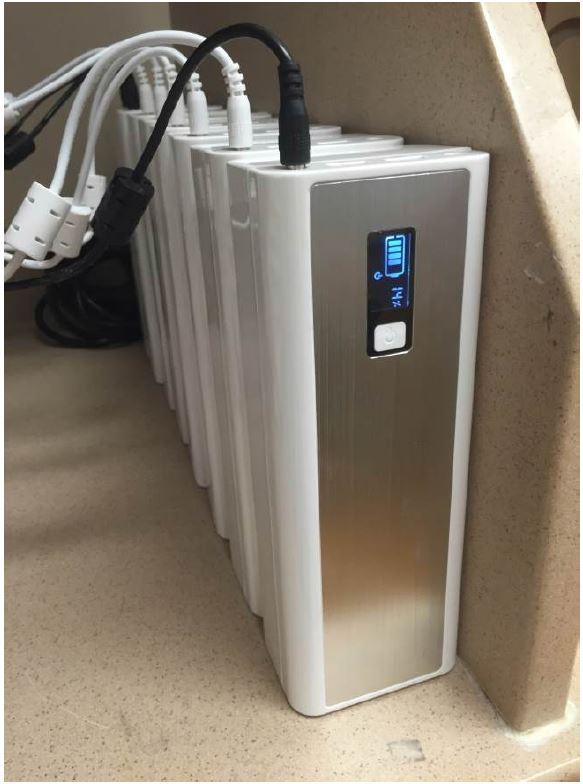
Multiple battery packs charging.

We opted for a system of exchangeable external batteries (Figure 2) rather than relying on the laptop’s internal battery to power the device. The internal battery lasts no more than 10 hours, while some ED shifts are as long as 12 hours. Because changing the internal battery would power down the machine, we chose a system of external batteries; during an external battery swap, the internal battery powers the VEAL, preventing shutdown. We purchased the *MP-50000 Powerbank, XTPower®* battery pack, which can power an active VEAL for over 12 hours per charge.

The implementation phase lasted six months, from September 2015 to February 2015. The ED staffed 27 attending and 14 resident physicians per month during this period, serving an average of 130 patients per day. Four to seven physicians are staffed at any given time. Five VEALs were initially introduced and six were available for use by the conclusion of the pilot period. Physicians were given the choice to use either the VEAL or the traditional workstations at their discretion. Traditional wall-mounted workstations were available in 60% of patient care rooms, and physicians had access to 10 dedicated workstations in the doctors’ charting room. VEAL usage was tracked via each laptop’s distinct IP address.

#### Committee Review

The study required no protected health information. All device usage data were collected in aggregate from the Allscripts^TM^ EHR system. All budgetary data were released freely without financial consideration.

## RESULTS

### Adoption

The standard monthly staffing level for this project’s ED requires 27 attending physicians and 14 resident physicians. In the first month of deployment, eight attending and three resident physicians adopted the five available VEAL units for clinical use; in the final month, adoption had increased to 12 attending and 10 resident physicians. Over the six-month implementation period, providers used the VEAL on 55 of the 130 patients (42.5%) treated per day on average in the ED, accessing each chart 12 times per patient visit. In the first month, the VEAL was used in the care of 35/138 (25.3%) patient visits per day with average chart access rate of 7.9 per visit. In the final month, those figures increased to 89/126 (70.6%) patients per day with average chart access rate of 14.0 times per patient visit.

VEAL adopters tended to be younger: 52% (14/27) of ED attending physicians had chosen to adopt the VEAL for clinical use, while resident physicians adopted the VEAL 78% (18/23) of the time.

### Cost

Cost data were obtained from supplier invoices. The podium and laptop were purchased for unit prices of $772 and $1,407 respectively. Two battery packs were purchased per VEAL for a combined cost of $459 per VEAL. Lastly, an *Imprivata®* ID badge reader for convenient EHR login access was added to each unit for $82 each. In total, each VEAL cost $2,721.

In comparison, our hospital’s COWs mobile carts were each purchased from Rubbermaid® at a price of ~$4,000.00 while the COW’s *Dell Optiplex 9020* PC and monitor cost ~$720. As with the VEAL, an Imprivata badge reader is affixed to each COW for $82. In summary, each COW costs a total of ~$4,857 or 78.5% more than each VEAL.

## DISCUSSION

We developed and implemented a smaller, more cost-effective and more functional mobile computing solution for the needs of a busy, academic ED with multiple providers and caregivers. Though initial adoption was faster by younger resident physicians, within six months of implementation, over 70% of all ED patients were cared for with this device. Adoption rates might have been slightly higher were adoption not restricted by the limited number of VEALs available during the pilot period. One indication of the demand for these units was the requests from other departments (e.g. trauma surgery, general surgery, otolaryngology) for identical units for their own use. We believe that several critical design features have contributed to the VEAL’s relatively rapid adoption and popularity despite its simple design.

While Wi-Fi is required for EHR use, it also augments patient care on several levels. First, it affords mobile conferencing services. This is particularly important for ASL, as The Joint Commission frequently cites hospitals for limitations in this area.^13^ Furthermore, telehealth can be particularly beneficial for healthcare centers without access to subspecialists. Because the VEAL can serve these multiple functions, dedicated telehealth, translation, and ASL units are unnecessary, saving both money and space. Second, the VEAL creates opportunities for patient engagement. Imaging and laboratory results can be accessed at the patient’s bedside for patient review. Similarly, accessing online videos, diagrams, and resources can augment real-time patient education.

Next, the exchangeable external battery system enables the VEAL’s continuous use. Since all mobile devices—from laptops to cellphones to bedside ultrasounds—are limited by battery life, dependability relies on a continuous power source. Unlike typical consumer electronic devices, which can be recharged while the user sleeps, rechargeable devices like COWs are limited for the 24/7 operations of the ED. In contrast, the VEAL’s power source is endlessly renewable.

**Figure 3:**
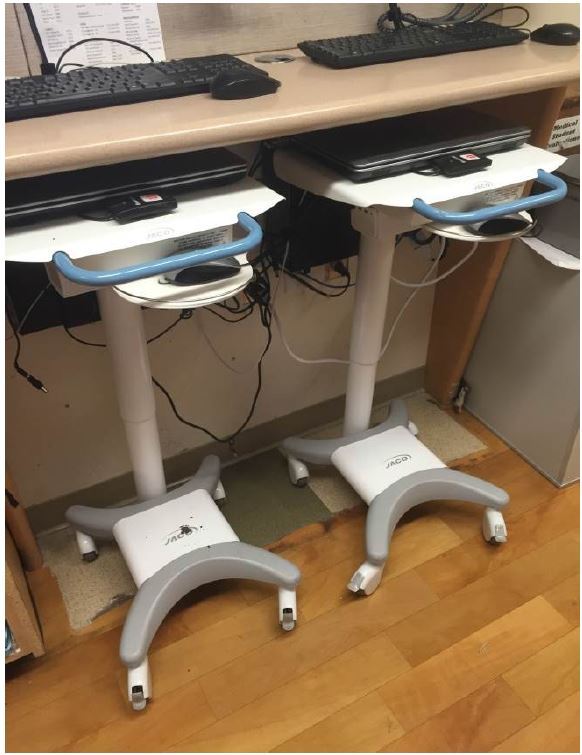
Very-Efficient Agile Laptop (VEAL) storage under physician work computers.

Most critical is the VEAL’s footprint. In addition to mobility, the VEAL’s small size allows it to be stored in our facility’s smallest corners (Figure 3) and allows patient care to extend to smaller treatment areas. Creating new patient care areas may be necessary as EDs struggle to cope with the well-documented and dramatic increases in ED patient volumes since the implementation of the Affordable Care Act of 2009.

For example, in many centers, physician-in-triage (PIT) systems have been implemented to expedite care and to quickly assign limited ED resources, such as gurneys, to patients who require true emergency care. In the study ED, VEAL’s mobility has been critical for the PIT system, which has two main treatment chairs, a single auxiliary bed, an isolation closet, and a hallway. The physician is able to treat and reevaluate multiple patients across a wide, yet cramped treatment area with a single mobile workstation.

One final advantage inherent to the VEAL is continuous sign-on. Because the VEAL stays with the provider throughout the work shift, the physician never needs to sign in or out of the EHR except at the beginning and end of the shift. Contrast this with the more common scenario of signing into or out of the nearest workstations. In the study ED, a provider spends 8–10 seconds per sign-in. In the study ED, providers average 20–25 patient encounters per 8–12 hour shift. With 13+ accesses per patient on average, the sign-on process with traditional workstations (not dedicated to a single physician) consumes some 40 minutes, and causes user frustration. Add to that the time spent walking the few feet each time to a stationary terminal, which also costs additional time and decreases productivity.

## LIMITATIONS

There are several limitations to this design and implementation study. The pilot population was small, limited to a single ED within an academic hospital. Results may not be directly applicable to a community hospital. Another limitation is that it was not possible to determine exactly what or how much patient care was performed on the VEAL (i.e. CPOE vs. documentation vs. bedside teaching). Lastly, secondary effects such as ED length of stay, patient satisfaction or quality of care were not examined during this pilot period. Long-term durability of the design and hardware is under scrutiny. We did not quantify VEAL use as a telemedicine or interpreter device.

## CONCLUSION

The VEAL is literally and metaphorically an example of leaner, more efficient healthcare. The VEAL delivers enhanced mobility and functionality at lower cost than its predecessor. Early performance data demonstrating rapid physician adoption and deployment in clinical care settings suggest a superior end-user experience. Additional study is needed to definitively demonstrate these benefits and their impacts on patient care. We are currently designing a study to quantify these effects. The VEAL may be an example of innovation improving care for both provider and patient.

As the needs of our patients and society evolve, we believe that the VEAL offers a leaner, higher-value healthcare experience than the COW of years past.
